# Neurocognitive Predictors of Response in Treatment Resistant Depression to Subcallosal Cingulate Gyrus Deep Brain Stimulation

**DOI:** 10.3389/fnhum.2017.00074

**Published:** 2017-02-24

**Authors:** Shane J. McInerney, Heather E. McNeely, Joseph Geraci, Peter Giacobbe, Sakina J. Rizvi, Amanda K. Ceniti, Anna Cyriac, Helen S. Mayberg, Andres M. Lozano, Sidney H. Kennedy

**Affiliations:** ^1^Department of Psychiatry, University Health NetworkToronto, ON, Canada; ^2^Faculty of Medicine, University of TorontoToronto, ON, Canada; ^3^Department of Psychiatry, St. Michael’s HospitalToronto, ON, Canada; ^4^Arthur Sommer Rotenberg Suicide and Depression Studies Program, St. Michael’s HospitalToronto, ON, Canada; ^5^Li Ka Shing Knowledge Institute, St. Michael’s HospitalToronto, ON, Canada; ^6^Krembil Research Institute, University Health NetworkToronto, ON, Canada; ^7^Department of Psychiatry and Behavioral Neuroscience, McMaster UniversityHamilton, ON, Canada; ^8^Department of Molecular Medicine and Pathology, Queen’s UniversityKingston, ON, Canada; ^9^Department of Pharmaceutical Sciences and Neurosciences, University of TorontoToronto, ON, Canada; ^10^Canadian Institute for Health InformationToronto, ON, Canada; ^11^London School of Hygiene and Tropical MedicineLondon, UK; ^12^Psychiatry and Behavioral Sciences, Emory UniversityAtlanta, GA, USA; ^13^Division of Neurosurgery, Department of Surgery, Krembil Neuroscience Centre, University Health NetworkToronto, ON, Canada

**Keywords:** treatment-resistant depression, cognition, deep brain stimulation, neurostimulation, neuropsychology, mood disorders

## Abstract

**Background:** Deep brain stimulation (DBS) is a neurosurgical intervention with demonstrated effectiveness for treatment resistant depression (TRD), but longitudinal studies on the stability of cognitive parameters following treatment are limited. The objectives of this study are to (i) identify baseline cognitive predictors of treatment response to subcallosal cingulate gyrus (SCG) DBS for unipolar TRD and (ii) compare neurocognitive performance prior to and 12 months after DBS implantation.

**Methods:** Twenty unipolar TRD patients received SCG DBS for 12 months. A standardized neuropsychological battery was used to assess a range of neurocognitive abilities at baseline and after 12 months. Severity of depression was evaluated using the 17 item Hamilton Rating Scale for Depression.

**Results:** Finger Tap-Dominant Hand Test and total number of errors made on the Wisconsin Card Sorting Test predicted classification of patients as treatment responders or non-responders, and were independent of improvement in mood. Change in verbal fluency was the only neuropsychological test that correlated with change in mood from baseline to the follow up period. None of the neuropsychological measures displayed deterioration in cognitive functioning from baseline to repeat testing at 12 months.

**Limitations:** This was an open label study with a small sample size which limits predictive analysis. Practice effects of the neuropsychological testing could explain the improvement from baseline to follow up on some tasks. Replication using a larger sample of subjects who received neuropsychological testing before and at least 12 months after DBS surgery is required.

**Conclusion:** These preliminary results (i) suggest that psychomotor speed may be a useful baseline predictor of response to SCG DBS treatment and (ii) support previous suggestions that SCG DBS has no deleterious effects on cognition.

## Introduction

Treatment resistant depression (TRD) occurs in 30% of depressed patients attending hospital clinics (Rush et al., 2006) and has a prevalence of 22% in Canadian community samples (Rizvi et al., 2014). Despite advances in drug development to treat major depressive disorder (MDD), there is no evidence that newer drugs have higher efficacy compared to first generation tricyclic antidepressants (Baghai et al., 2011). On the other hand, functional neuroimaging research has provided evidence for abnormal neuronal circuits in MDD (Mayberg et al., 1999) which has contributed to the emerging interest in deep brain stimulation (DBS) and other targeted neurostimulation techniques to provide alternative treatment options for TRD.

Deep brain stimulation is an experimental neurosurgical procedure that was initially developed for use in movement disorders (Deep Brain Stimulation for Parkinson’s Disease Study Group, 2001) as well as advancing knowledge about neuro-circuitry dysfunction in relation to depression (Russo and Nestler, 2013). The first DBS study in TRD to modulate subcallosal cingulate gyrus (SCG) over-activity was published in Mayberg et al. (2005), and subsequent open-label trials have demonstrated that it is safe and effective in small open-label trials for treatment of TRD (McNeely et al., 2008; Holtzheimer et al., 2012; Merkl et al., 2013; Bogod et al., 2014). A recent systematic review of DBS for TRD including a range of stimulation targets reported an overall response rate of 40–70% (Morishita et al., 2014).

The underlying mechanism of SCG DBS is not yet fully understood, but is thought to cause both local and distributed effects through its modulation of the mood-regulatory network that is considered dysregulated in MDD (Mayberg et al., 2005). In previous pharmacologic and non-pharmacologic treatment studies of depression (Mayberg et al., 1999, 2000; Goldapple et al., 2004), clinical response appears to best correlate with limbic/paralimbic decreases involving the orbital frontal cortex and subgenual cingulate regions (Mayberg et al., 2005). Based on these findings, we hypothesized that DBS to the subcallosal region would result in deactivation of the local SCG (BA25) region and normalization of hypoactive cortical regions. We further hypothesized that this combination of limbic-paralimbic decreases and dorsal cortical increases are necessary for clinical remission (Mayberg, 2003; Mayberg et al., 2005).

Cognitive deficits are present in the majority of patients with MDD (McIntyre et al., 2013), and these deficits are not necessarily reversed by antidepressants (Biringer et al., 2007; Baune et al., 2010; Godard et al., 2012). To address concerns that neurosurgery may worsen or create new cognitive deficits, it is important to measure the effects of DBS on cognition and explore cognition indices as potential predictors of DBS response. Stimulation of the SCG could impact neurocognitive performance through its direct connections to the nucleus accumbens, amygdala, ventral striatum and prefrontal cortex, regions involved in the dopaminergic networks associated with psychomotor processing (Johansen-Berg et al., 2008).

The effects of SCG DBS on cognition have been evaluated in four smaller longitudinal studies, although none have evaluated baseline neurocognitive measures as moderators of response or non-response to DBS (McNeely et al., 2008; Holtzheimer et al., 2012; Merkl et al., 2013; Bogod et al., 2014). Moreines et al. (2014) reported that TRD (mix of unipolar and bipolar) patients had poorer performance than healthy controls on processing speed tasks at baseline but following SCG DBS, there was no deterioration of neuropsychological function and in fact improvement in processing speed and executive function after 6 months occurred. Similarly, another SCG DBS for TRD study found no deterioration of cognitive functioning following 1 year of stimulation and no relationship was found between depression rating and cognitive testing (Serra-Blasco et al., 2015). The purpose of the present study was to investigate the effect of DBS on cognition and to determine whether cognitive indices at baseline predicted DBS outcome.

## Materials and Methods

### Participants

Details of the trial methodology have been published previously (Mayberg et al., 2005; Lozano et al., 2008). Twenty TRD patients were enrolled in a 12 month open label trial of SCG DBS between 2003 and 2006, during which no new psychotropic medications were added. Response was defined as 50% or greater reduction in the 17-item Hamilton Rating Scale for Depression (HRSD-17) 12 months after surgery (Hamilton, 1960). The selection criteria for patients have also been reported in a previous paper (Lozano et al., 2008). Referrals came from hospital and community psychiatrists who were aware of the protocol and were not directly involved in its implementation. All patients met criteria for a more intractable form of TRD (see below) and were in a current major depressive episode (MDE) for a minimum of 1 year with a minimum score of 20 on the HRSD-17. Research Ethics Board approval and informed consent were obtained as previously outlined (Mayberg et al., 2005).

### Inclusion and Exclusion Criteria

For the protocol, treatment resistance was defined as failure to respond to a minimum of four different treatments including antidepressant pharmacotherapies of sufficient dose and duration, evidence-based psychotherapy and ECT (unless otherwise contraindicated). Exclusion criteria included comorbid Axis I psychiatric disorders (with the exception of Generalized Anxiety Disorder), a cluster B Axis II diagnosis as determined by the Structured Clinical Interview for DSM-IV Axis II Personality Disorders (SCID-II) (American Psychiatric Association, 2000), suicide attempt within the past year or a score of 3 or more on the HRSD-17 suicide item, or an unstable medical condition.

### Surgery and Stimulation Settings

Deep brain stimulation electrodes were implanted in SCG white matter under local anesthesia using magnetic resonance (MR) imaging guidance. Monopolar stimulation was used at 90us pulse width and 130 Hz. Voltage was adjusted to a maximum of 9.0 V at each of the eight electrode contacts based on effectiveness and tolerability (Lozano et al., 2008). Stimulation parameters were adjusted at follow-up visits based on symptom improvement or adverse effects, with patients receiving stimulation between 3.5 and 5.0 V.

### Neuropsychological Assessment and Study Protocol

A battery of neuropsychological tests was administered at baseline (before surgical implantation) and 12 months post-operatively. The neuropsychological tests conducted have been described in an earlier study on a smaller sample of this cohort (McNeely et al., 2008) and are listed in **Table 1** with the dependent variables for each neuropsychological test illustrated. The neuropsychological assessment battery was designed to assess general cognitive performance as well as detailed frontal lobe functioning. The test battery was carefully designed to differentiate dorsolateral, superior medial, and ventrolateral/orbital frontal cognitive functions, as it was anticipated at the outset of the study that different frontal regions may be differentially affected by activation or disruption of SC tracts by chronic DBS. To address re-test learning effects, alternate versions of neuropsychological tests were used when available.

**Table 1:** Neuropsychological tests.

Assessment	Test	Dependent variable
Executive Function	Wisconsin Card Sort Test (WCST)	•Number of categories completed •Total number of errors
Verbal Learning and Memory	Hopkins Verbal Learning Test (HVLT)	•Total word recall over three trials
Verbal Fluency	Controlled Oral Word Association Test (COWA)	•Total number of words
Processing Speed	Finger Tap Test	•Mean of five consecutive 10 s trials
Attention	Stroop Test	•Stroop word, color, and interference

### Statistical Analysis

The Statistical Package for the Social Sciences (SPSS), Edition 20 for Windows was used (IBM Corp. Released, 2011). Scores were corrected for age, sex and education and converted into z-scores in order to assess patients’ performance relative to a normative population (Strauss et al., 2006). A z-score greater than 1 standard deviation (SD) below the norm was interpreted as below average and z-scores more than 1 SD above the norm were above average, in keeping with profile interpretation methods used in other psychiatric populations (Matsui et al., 2007; Kim et al., 2009). We conducted the Shapiro–Wilk test and examined QQ plots to assess whether data were normally distributed. We proceeded to carry out Student’s *t*-test analysis on all normally distributed data from the completed neuropsychological tests. Where data were not normally distributed, the non-parametric Mann–Whitney *U*-test was performed. Significance of change between baseline and 12 months was analyzed via paired *t*-test for each neuropsychological test.

## Results

### Demographic and Clinical Characteristics

Patient demographic and clinical characteristics of responders and non-responders are shown in **Table 2**. All patients had received psychotherapy and 85% (*n* = 17) had received ECT, with eight patients (47%) having had a clinical response to ECT. 55% (*n* = 11) of the patients were female, with an average age of 47 years (SD 10) and range 29–71 years. The average duration of MDE was 6.9 years (SD 5.6). Patients had a NAART mean estimated Full Scale IQ score of 110.8 (SD 8.5) which was reflective of general or above normal range intelligence. The average HRSD-17 score at baseline was 24.3 (SD 3.5) and at follow up was 12.3 (SD 6.6).

**Table 2:** Demographic and clinical characteristics of patient cohort.

	*N*	%
**Sex**		
Male	9	45
Female	11	55
Past ECT	17	85

	**Mean**	***SD***

Age (years)	47.4	10.4
Duration of MDE (years)	6.9	5.6
Lifetime Number of MDE	3.9	3.1
Number of Medications	4.2	4.1
HRSD Baseline	24.3	3.5
HRSD 12 Month	12.3	6.6
NAART-IQ	110.8	8.5

Patients were classified as responders or non-responders after 12 months of chronic SCG DBS. There were 11 responders (55%) and 9 non-responders (45%). There were no significant differences between responders and non-responders in relation to age, gender, duration of depressive episode or number of depressive episodes. Baseline HRSD-17 scores were not significantly different between responders and non-responders, *t*(18) = 0.11, *p* = 0.92. There was a significant reduction in HRSD-17 scores from baseline to follow up, *N* = 20, *t*(19) = 8.5, *p* = 0.001.

At the time of surgery, patients were receiving mean 4.2 medications (median 4). Two patients were only receiving a benzodiazepine but had discontinued all antidepressant medications, two patients were receiving only one antidepressant, five patients were receiving an antidepressant augmented with an antipsychotic agent or benzodiazepine, and 11 patients were receiving two antidepressants from two different classes, combined with lithium or an atypical antipsychotic and a benzodiazepine. Attempts were made to keep patients’ medications constant throughout the study, so as to reduce confounders to treatment response. Four patients (20%) had their antidepressant dosage reduced and all of these patients were responders. One patient had the dose of antidepressant treatment increased (from citalopram 20 to 30 mg) at the 6 month period and this patient was a non-responder.

### Neuropsychological Performance at Baseline and 12 months

The mean North American Adult Reading Test (NAART) for this sample was 1 SD above the mean (see **Table 2**). Using profile interpretation methods in this sample whose pre-morbid cognitive function would be expected to fall 1 SD above the mean corresponding to general intellect, we found 1 SD below the normative mean to be a reasonable cut-off for clinically significant relative cognitive impairment. All patients completed neuropsychological testing at baseline. After 12 months, four of the twenty patients were unavailable for neuropsychological retesting; two had the device explanted, one left the country and one was lost to follow up. The remaining 16 patients as a group displayed no evidence of deterioration of cognitive functioning over the 12 month follow up period (see **Table 3**). At 12 months, performance on the Wisconsin Card Sorting Task (WCST) improved significantly in three of the four subscales (see Supplementary Table S1).

**Table 3:** Neuropsychological test results from baseline to 1 year follow up.

Scale	Baseline Mean (*SD*)	1 Year Mean (*SD*)	*t*-test	*p*	Baseline Z	1 Year Z	Change Mean
**Finger Tap (*n* = 13)**							
Dominant Hand	44.4 (10.8)	47.5 (5.8)	-1.2	0.23	-0.4	-0.3	+0.1
Non-Dominant Hand	39.8 (8.6)	43.1 (6.8)	-1.8	0.08	-0.59	-0.24	+0.35
**Hopkins Verbal Learning Test (*n* = 14)**	25.3 (4.4)	27.3 (4.6)	-2.2	0.06	-0.63	-0.41	+0.22
**Verbal Fluency (*n* = 13)**	43.3 (14.5)	44.1 (13.5)	-0.16	0.87	-0.14	0.07	+0.21

**FIGURE 1:**
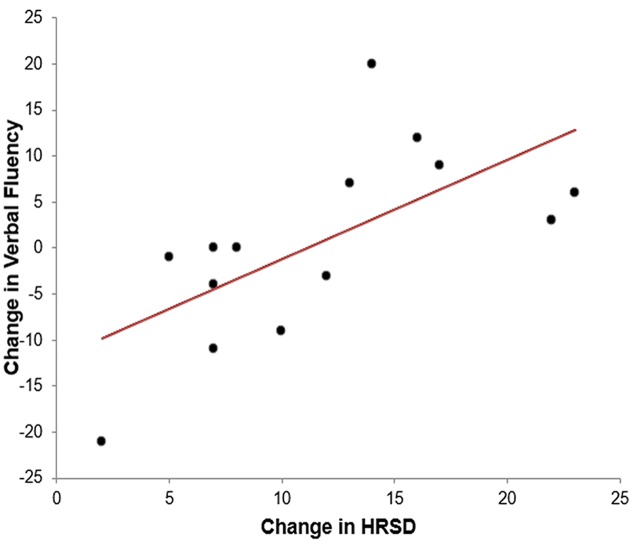
**Scatterplot of change in HRSD and change in verbal fluency**.

Not all neuropsychological data were captured at the 12 month follow up period. Therefore, **Table 3** includes the number of patients who completed each test at both baseline and follow up. At baseline, there was clinically significant impairment in the total sample (*Z* = -1 SD below the normative mean) in information processing/attentional speed (Stroop color reading, color-word speed; see Supplementary Table S2) and executive functioning (WCST category score and number of perseverative and non-perseverative errors). There was no impairment relative to normative data in psychomotor speed (finger tapping), verbal memory [Hopkins Verbal Learning Test (HVLT)] or verbal fluency [Controlled Oral Word Association Test (COWA)]. There was no statistically significant deterioration in cognitive functioning on any of the tests over the 12 month follow up period. Paired *t* -test revealed significant improvements (*p* < 0.05) in executive function (WCST category score, total number of errors, perseverative but not non-perseverative errors) and approached significant improvement on verbal memory (HVLT recall) (*p* < 0.06). There was no deterioration in psychomotor speed (finger-tapping test) (see **Table 3**). The only neuropsychological variable associated with change in HRSD-17 score over the 12 month period was verbal fluency [*r*(13) = -0.63, *p* < 0.01], such that improvement in verbal fluency was associated with a larger change in depression rating (see **Figure 1**).

### Responder-Non-responder Differences in Neuropsychological Testing

Baseline scores differed significantly between responders and non-responders on the WCST -Total errors (*U* = 19, *Z* = -2.1, *p* = 0.035) and Finger Tapping Test scores with both the dominant hand [*t*(18) = -2.5, *p* = 0.03] and non-dominant hand (*U* = 15, *Z* = -2.6, *p* = 0.007). Correlations were carried out between each of these tests and baseline HRSD-17 scores (*n* = 20). No correlation was found between WCST -Total errors and HRSD-17 scores at baseline [*r*(18) = -0.05, *p* = 0.85]. Similarly, there was no correlation between depression scores and scores on either the dominant [*r*(19) = -0.26, *p* = 0.28] or non-dominant [*r*(19) = 0.09, *p* = 0.7] hand Finger Tap Test.

### Predictors of Response to DBS

We conducted *t*-test analyses to examine which baseline neuropsychological variables discriminated responders from non-responders. Three variables were found to have a statistically significant difference between responders and non-responders. **Table 4** shows the performance of responders and non-responders bilaterally on the Finger Tap Test and WCST -Total errors in mean values following *t*-test analysis.

**Table 4:** Summary of *t*-test of baseline responders to DBS at 1 year follow up.

Test	Mean	*SD*	*T*	*p*
**Finger Tap Dominant Hand**				
Responder	50.1	6.8	-2.5	0.03
Non-Responder	39.3	12.2		
**Finger Tap Non-Dominant Hand**				
Responder	44.2	4.0	*Z* = -2.6	0.007
Non-Responder	34.9	8.9	Non-Parametric (Mann–Whitney U)	
**WCST Total Errors**				
Responder	52.0	12.4	-2.1	0.048
Non-Responder	41.3	8.9		

Despite our small sample size of 20 (11 responders and 9 non-responders), we utilized machine learning protocols as a proof of concept exercise to test a model including WCST-Total errors, Finger Tap Dominant Hand and Non-Dominant Hand for prediction of response to DBS treatment. Two of these variables, WCST-Total errors and Finger Tap Dominant Hand were selected via a feature selection (FS) protocol (Zhao et al., 2011).

Cross-validation (CV) has been proposed in situations where an attempt at a true replication would be premature (Kohavi, 1995). This process allows one to construct a model on a portion of data while testing it on a portion that has been left out. For the models reported here we use a 10-fold cross-validation, though other proportions were checked and the results remained consistent. In a 10-fold validation, 18 patients are chosen to be modeled and two are left out to be tested. The overall reported accuracy reflects the performance of this process for the aforementioned variables.

**FIGURE 2:**
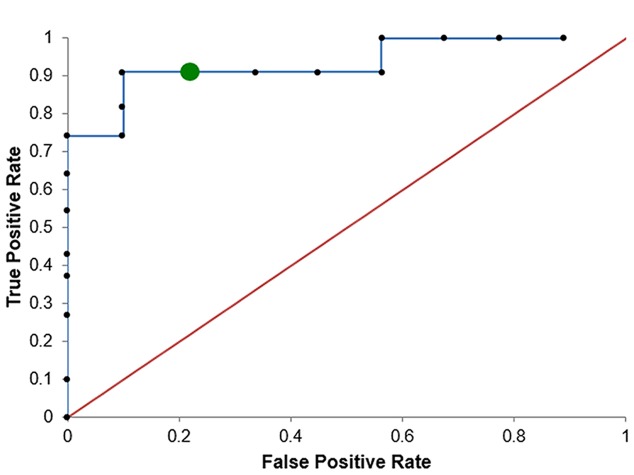
**ROC for neural networks**.

The artificial neural networks (NNs) machine learning method was utilized in this analysis (Azimi et al., 2014). NNs have the benefit of being able to capture non-linear relationships between variables and are able to handle noisy data, although they can be prone to over-fitting and the models are often difficult to interpret (Azimi et al., 2014). The results of our model are presented in **Table 5** and **Figure 2**.

**Table 5:** Tabulation of the ROC for neural networks model.

	Predicted		
	Non-Responder	Responder	Error
**Actual**			
Non-Responder	8	1	1/10
Responder	1	10	1/9

Utilizing WCST Total errors and Finger Tap Dominant Hand, this 10-fold CV for the artificial neural net only misclassified patients 10% of the time. The model itself was able to predict 8/9 non-responders and 10/11 responders correctly from our population of patients. The area under the curve (AUC) of the cross-validation procedure was found to be 92.9%.

## Discussion

This longitudinal study of cognitive functioning in TRD patients who have undergone SCG DBS provides evidence for the stability of cognitive functioning at least 1 year after surgery. Despite this use of long-term neurostimulation, there is no evidence of any acquired or accumulated cognitive dysfunction. Indeed, any improvements in cognitive function over the 12 month period were independent of improvements in mood, with the exception of verbal fluency. The present findings of cognitive stability following DBS are consistent with previous findings. A previous report on six patients from this cohort also showed no deterioration in cognition, and most patients’ performance improved from the clinically impaired range at baseline to the average range at follow up (McNeely et al., 2008). This is in contrast to the well-documented association between cognitive impairment and ECT (Kellner et al., 2010).

Our findings are also in agreement with an extended report on four patients who were reassessed for up to 42 months after surgery (Bogod et al., 2014). Two additional longitudinal studies of inpatient MDD samples have previously demonstrated that improvement in verbal fluency was correlated with improvement in mood on hospital discharge (Trichard et al., 1995; Neu et al., 2001).

Evidence from a Positron emission tomography (PET) study (Gourovitch et al., 2000) showing left frontal lobe activation during a verbal fluency task [including dorsolateral prefrontal cortex (DLPFC) and anterior cingulate] supports a link between mood and activation of the DLPFC, the anterior cingulate, or indeed an interaction of both regions. Explicit memory deficits in MDD patients were found to be were independent of current (state-related) mood, while the number of past depressive episodes (trait-related) determined the hippocampal-dependent cognitive deficits (MacQueen et al., 2002). The absence of any other correlations between changes in cognitive measures and mood raises the possibility that certain areas of the brain assessed by such tests may be more sensitive to clinical state, while other functions are more likely to be trait-related.

### Cognitive Changes in Responders Relative to Non-responders

As far as we are aware, this is the first study to explore baseline prediction of response to SCG DBS treatment from neurocognitive measures. Dominant-hand finger tap test and WCST-Total errors predicted treatment response with a high degree of accuracy. The WCST-Total errors score reflects the sum of both perseverative errors (indicative of inability to shift mental set in response to feedback) and non-perseverative errors (indicative of loss of mental set, random responding, or conceptual inability). While the WCST has long been held as a gold standard measure of prefrontal function, with perseverative errors considered the main index of frontal dysfunction and number of conceptual responses also indicated, impaired WCST performance in patients with frontal dysfunction reflected both perseverative and random errors (Lie et al., 2006). Only WCST Total Errors, and not the other outcome variables of this test (number of categories completed, perseverative or non-perseverative errors alone), were predictive of response to treatment in TRD patients, suggesting that prefrontal function at baseline may be driving this relationship. The anterior cingulate may be particularly important, as this region of prefrontal cortex is implicated in error detection during WCST performance, though it appears that a widely distributed frontal-posterior network is required for overall successful performance of the many cognitive processes involved in the WCST (Nyhus and Barceló, 2009). Improvement on the Finger Tap Test, a measure of psychomotor speed, has support in the literature as a marker of treatment response, whereby patients who achieved remission had significantly less baseline psychomotor dysfunction than subsequent non-remitters (Gallagher et al., 2007). In our study, although baseline finger tap testing did not correlate with change in mood over the follow up period in the sample as a whole, the dominant hand finger tap discriminated responders from non-responders, suggesting that psychomotor speed may be an independent predictor of treatment response and a potential biomarker to evaluate pre-treatment outcome. A realistic interpretation of these results is that the variables in this model are predictive for our population and would be worth exploring in a larger DBS cohort considering the small sample size. These models reinforce the hypothesis that it is possible to predict response to DBS treatment and that the WCST Total Errors and Finger Tap Dominant Hand scores may play an informative role.

Psychomotor symptoms are regulated by dopamine rich striatal brain regions important for motor control (Meyer et al., 2006) and have been shown to predict response to antidepressant medications (Caligiuri et al., 2003; Herrera-Guzmán et al., 2008). This is supported by reports of patients with greater psychomotor retardation demonstrating higher D2 binding (Ebert et al., 1996; Meyer et al., 2006), as well as correlations between D2 binding and symptom severity (Larisch et al., 1997; Lehto et al., 2008). Therefore, subsequent non-responders to SCG DBS may exhibit greater deficits than responders in dopaminergic symptoms such as anhedonia and psychomotor retardation. The SCG has direct connections to areas that are involved in the dopaminergic networks implicated in psychomotor processing such as the ventral striatum, nucleus accumbens, the amygdala and the prefrontal cortex (Johansen-Berg et al., 2008). Our finding that reduction in the severity of psychomotor retardation predicts response to DBS suggests that SCG DBS may have a positive effect on dopamine function. An alternate explanation of these findings is that DBS to SCG may also impact activity of the supplementary motor area, which plays a role in psychomotor retardation. Modulation of this region may represent another pathway to mediating changes in willed action that are demonstrated through neurocognitive tests such as the Finger Tap Test.

Further support for the role of psychomotor retardation in depression comes from a Single Photon Emission Computerized Tomography (SPECT) study, in which dopamine D2 binding in MDD patients was positively correlated with motor speed and negatively correlated with verbal fluency, independent of mood rating (Shah et al., 1997). Changes in the plasma levels of dopamine precursors correlated with HRSD-17 scores, cognitive disturbance and retardation factors (Martinot et al., 2001). Three functional imaging studies clearly demonstrated a striatal dopaminergic disturbance during depression, which was most prominent when patients displayed motor retardation (Shah et al., 1997; Martinot et al., 2001; Meyer et al., 2006), corroborating findings of therapeutic effects of dopaminergic drugs in depression associated with psychomotor symptoms (Mann and Kapur, 1995).

### Limitations

Despite the generally positive findings regarding cognitive outcomes, certain limitations of the study must be acknowledged. This was an open label study with a small sample size which thereby limits the predictive analysis. While the current findings are promising, replication in a larger sample is necessary in order to establish a reliable neurocognitive predictor of response to DBS. In addition, the use of multiple two-sample *t*-test due to the small sample size should be acknowledged as a limitation. Practice effects must also be taken into account and may have contributed to some extent to the improvements noted at the 12 month follow-up. While attempts to minimize practice effects were in place, including selecting memory and verbal fluency tasks with alternate versions, as well as other tasks known to be less sensitive to practice after long test-retest intervals (e.g., Finger Tap, Stroop Test), non-specific and test specific practice effects could explain the improvement from baseline to follow up on some tasks. However, these potential practice effects do not account for the predictive nature of the finger tap test result that discriminates responders from non-responders. While there was no correlation between dose change and neuropsychological performance, the exact role played by medication change is uncertain. The modeling methods used in this paper are a preliminary proof of concept that would usually be used for studies with a larger sample size.

The strengths of the study lie in its longitudinal design and the fact that there remain few studies on the longitudinal neuropsychological effects of DBS for TRD. The current results suggest that most cognitive deficits in this population are separate from the impact of mood, and that the SCG DBS treatment may also provide positive benefits in executive and motor functioning to those suffering from TRD. Finally, the present findings are the first to demonstrate baseline cognitive performance as a preliminary predictor of treatment response with SCG DBS. There appears to be converging evidence that psychomotor speed may be a viable predictor though due to the limitations described above, the findings in this study would benefit from replication in an adequately powered sample.

## Ethics Statement

University Health Network Research Ethics Board, Toronto, ON, Canada. Referrals were made by psychiatrists not affiliated with the study. Following referral, potential subjects were seen by two independent psychiatrists to assess study eligibility. If potentially eligible at that point, discussions took place between the study team and potential participant. Potential subjects were encouraged to attend these further discussions with a family member. Following discussions with one of the principal investigators, interested individuals were then referred to the neurosurgeon (AL), who provided details on the surgical procedure. At that point, interested candidates were provided with informed consent forms to review and discuss further with the study team or family members as needed. Approximately 6–8 weeks elapsed between initial referral and study consent.

## Author Contributions

All authors have materially participated in the research and/or article preparation. SM completed the statistical analysis of the data and wrote the manuscript. HEM conducted the neuropsychological testing, advised on the analysis of the data and assisted with the preparation of the article for publication. JG assisted with the statistical analysis and preparation of the article. PG was involved in the study design and the assessment of the patients recruited for the study, and advised on the preparation of the article. SR was the coordinator for the original study and she assisted with the preparation of the article. AC provided input into the acquisition of the data and assistance with the formatting of the article. AKC contributed to preparation and revision of the article for publication. HSM contributed to the conception and design of the study. AL contributed to the conception and design and conducted the neurosurgical procedures. SK contributed to the conception and design of the study, advised on the interpretation of the data, assisted significantly to the drafting of the article and revised it critically for important intellectual content. All co-authors gave final approval of the final article.

## Conflict of Interest Statement

PG has been a consultant for St. Jude Medical. HSM has received consulting and intellectual licensing fees from St. Jude Medical, Inc. AL has been a Consultant for Medtronic, St. Jude Medical and Boston Scientific. He has intellectual property in the field of DBS. SK has received grant/research support from Ontario Brain Institute (OBI) and CIHR. He has received research support from Lundbeck, St. Jude Medical, Bristol-Myers Squibb, and Clera, Inc. He has received speaking fees from and/or is a member of an advisory board for: Lundbeck, Lundbeck Institute, Pfizer, Bristol-Myers Squibb, Servier, and Forest, Janssen Pharmaceutical Companies of Johnson and Johnson. SMI received a CANMAT-Pfizer fellowship during the time this analysis was completed. SM, PG, AKC, SR, and SK have contributed to the CAN-BIND (Canadian Biomarker Integration Network in Depression) Program which is supported by the OBI. AC, HEM, and JG have no conflicts of interest.
