# Supplementary material for: Neurocognitive Predictors of Response in Treatment Resistant Depression to Subcallosal Cingulate Gyrus Deep Brain Stimulation
Source: Front Hum Neurosci. 2017 Feb 24;11:74. doi: 10.3389/fnhum.2017.00074 (PMC5323405; doi:10.3389/fnhum.2017.00074)
Supplement: Supplementary file 1 [file Table_1.DOCX]

Supplementary Material

Neurocognitive Predictors of Response in Treatment Resistant Depression to Subcallosal Cingulate Gyrus Deep Brain Stimulation

Shane J. McInerney, Heather E. McNeely, Joseph Geraci, Peter Giacobbe, Sakina J. Rizvi, Anna Cyriac, Amanda K. Ceniti, Helen S. Mayberg, Andres M. Lozano, Sidney H. Kennedy^*^

*** Correspondence:** Sidney H. Kennedy: sidney.kennedy@uhn.ca

# Supplementary Tables

**Supplementary Table 1** Wisconsin Card Sorting Test (WCST) Results from Baseline to One Year Follow Up (n=13)

| Scale | Baseline Mean (SD) | 1 Year  Mean (SD) | t-Test | | Baseline Z | 1 Year Z | Change Mean |
| --- | --- | --- | --- | --- | --- | --- | --- |
|  |  |  | **t** | ***p*** |  |  |  |
| WCST T Scores |  |  |  |  |  |  |  |
| Category | 29.9 (19.4) | 46.0 (10.1) | -3.6 | 0.006 | -2.1 | -0.4 | +1.7 |
| Total Errors | 40.3 (6.2) | 50.6 (8.2) | -5.2 | 0.001 | -0.97 | 0.06 | +1.03 |
| Non-Perseverative Errors | 38.4 (7.6) | 51.8 (12.2) | -3.0 | 0.01 | -0.86 | 0.03 | +0.89 |
| Perseverative Errors | 42.0 (3.8) | 46.3 (11.2) | -1.3 | 0.22 | 0.92 | 0.06 | +0.14 |

**Supplementary Table 2** Stroop Test Results from Baseline to One Year Follow Up (n=13)

| Scale | Baseline Mean (SD) | 1 Year  Mean (SD) | t-Test | | Baseline Z | 1 Year Z | Change Mean |
| --- | --- | --- | --- | --- | --- | --- | --- |
|  |  |  | **t** | ***p*** |  |  |  |
| Stroop Word | 88.7 (22) | 94.3 (19.2) | -1.3 | 0.21 | -0.97 | -0.21 | +0.76 |
| Stroop Color | 63.9 (14.4) | 68.8 (18.5) | -1.4 | 0.18 | -1.0 | -0.59 | +0.41 |
| Stroop Word-Color | 38.4 (11.7) | 41.5 (12.6) | -1.4 | 0.19 | -0.64 | -0.35 | +0.29 |
| Stroop Interference | 1.92 (7.7) | 2.23 (6.0) | -0.21 | 0.84 | 0.19 | 0.22 | +0.03 |
